# Frequency of adding salt at the table and risk of incident cardiovascular disease and all-cause mortality: a prospective cohort study

**DOI:** 10.1186/s12916-022-02691-9

**Published:** 2022-12-15

**Authors:** Fengping Li, Liangkai Chen, Buyun Liu, Victor W. Zhong, Yan Deng, Dan Luo, Chao Gao, Wei Bao, Shuang Rong

**Affiliations:** 1 Department of Nutrition, School of Public Health, Wuhan University, Research Center of Public Health, Renmin Hospital of Wuhan University, No.115 Donghu Road, Wuhan, 430071 China; 2 Department of Nutrition and Food Hygiene, Hubei Key Laboratory of Food Nutrition and Safety, Ministry of Education Key Lab of Environment and Health, School of Public Health, Tongji Medical College, Huazhong University of Science and Technology, Wuhan, 430030 China; 3 Institute of Public Health Sciences, Division of Life Sciences and Medicine, University of Science and Technology of China, Hefei, 230026 China; 4 School of Public Health, Shanghai Jiao Tong University School of Medicine, Shanghai, China; 5 Academy of Nutrition and Health, Hubei Province Key Laboratory of Occupational Hazard Identification and Control, School of Public Health, Wuhan University of Science and Technology, Wuhan, 430065 China; 6 School of Nursing, Wuhan University, Wuhan, China; 7 Key Laboratory of Trace Element Nutrition of National Health Commission, National Institute for Nutrition and Health, Chinese Center for Disease Control and Prevention, Beijing, 100050 China

**Keywords:** Adding salt at the table, Cardiovascular disease, Mortality

## Abstract

**Background:**

Adding salt at the table is a prevalent eating habit, but its long-term relationship with cardiovascular disease (CVD) and all-cause mortality remains unclear. We evaluated the associations of adding salt at the table with the risk of incident CVD and all-cause mortality.

**Methods:**

Among 413,109 middle- and old-aged adults without cancer or CVD, all participants reported the frequency of adding salt at the table at baseline. The associations between adding salt at the table and incident CVD (the composite endpoint of coronary heart disease, stroke, heart failure, and CVD deaths) and all-cause mortality were investigated using Cox proportional hazards models.

**Results:**

Of the study population, the mean age was 55.8 years and 45.5% were men; 44.4% reported adding salt at the table; 4.8% reported always adding salt at the table. During a median follow-up of 12 years, there were 37,091 incident CVD cases and 21,293 all-cause deaths. After adjustment for demographic, lifestyle, and cardiometabolic risk factors, the multivariable-adjusted hazard ratios (HRs) for participants who always added salt at the table versus never/rarely added salt at the table were 1.21 (95% confidence interval [CI]: 1.16-1.26) for CVD, 1.19 (95%CI: 1.05–1.35) for CVD mortality, and 1.22 (95%CI: 1.16–1.29) for all-cause mortality, respectively.

**Conclusions:**

In this prospective cohort study, a higher frequency of adding salt at the table was associated with a greater risk of incident CVD and mortality. Our findings support the benefits of restricting the habit of adding salt at the table in promoting cardiovascular health.

**Supplementary Information:**

The online version contains supplementary material available at 10.1186/s12916-022-02691-9.

## Background

Cardiovascular disease (CVD) remains the leading cause of death worldwide. The Global Burden of Disease study has demonstrated that diet contributes significantly to the risk of CVD [1]. Although high sodium intake is closely related to the occurrence and development of CVD [2], many people have the habit of adding salt to their food before or after tasting it. Two studies using large nationally representative samples of households in the UK estimated that the proportion of habitually adding salt at the table was 31.7% and 40.2% [3, 4]. Evidence from nationwide data indicated that more than 40% of various racial/ethnic groups reported habitually adding salt at the table in the USA [5].

Preference or habit may prompt people to add discretionary salt to their food during cooking or at the table [6], which may be one of the main contributing mechanisms for long-term high salt consumption [7]. A recent systematic review reported that the average daily salt intake of adults worldwide ranged from 5.2 to 15.5 g/day [8], exceeding the recommended limit from the World Health Organization (WHO) [9] and current dietary guidelines [10]. Adding salt at the table may increase salt intake and remains a significant source of total salt intake, accounting for about 15–20% [4]. It was estimated that about 1.4 g of salt was added at the table or during cooking in the UK [11]. Another survey based on Samoan surveillance data found that people who added salt at the table had 1.5 g higher salt intake than those who did not [12]. However, evidence on the relationship of adding salt at the table with the risk of incident CVD and mortality is limited. One study, including 11,814 adults aged 46–66, showed that adding salt at the table was associated with a 15% higher risk of heart failure [13]. Another study, including 11,742 community older men, found that always adding salt to food was associated with a 12% higher risk of all-cause mortality [14]. Moreover, the long-term health effect of adding salt at the table on incident CVD and all-cause mortality remains unclear in the general population.

Therefore, the objective of this study was to examine the associations of the frequency of adding salt at the table with incident CVD, CVD mortality, and all-cause mortality in a prospective cohort study of nearly half a million adults.

## Methods

### Study design and participants

Between 2006 and 2010, UK Biobank recruited over 500,000 middle- and old-aged UK adults [15]. Individuals registered with the National Health Service and living within 25 miles of a UK Biobank assessment center were invited to participate voluntarily. Of the 9 million individuals invited to participate, 5.5% were ultimately enrolled. All participants attended 1 of 22 assessment centers where they completed questionnaires, took physical measurements, and provided biological samples [15]. All participants provided informed consent through electronic signature at baseline assessment, and the UK Biobank study was approved by the North West-Haydock Research Ethics Committee (16/NW/0274). Participants who withdrew from UK Biobank (*n* =46) and had cancer (*n* =56,029) or CVD (*n* =31,637) at baseline and those without data on salt added at the table (*n* =956) and those who died from coronavirus disease 2019 during pandemic (*n* =728) were excluded from the analysis. The final analytical sample included 413,109 participants (Additional file 1: Fig. S1).

### Exposure assessment

At the baseline, the frequency of adding salt at the table was recorded. Participants were asked to report their daily salt use habits, with the question “Do you add salt to your food? (Do not include salt used in cooking)”. Possible responses include “Never/rarely,” “Sometimes,” “Usually,” or “Always.” Discretionary salt is usually added during cooking or at the dinner table [3]. Therefore, adding salt to food other than during cooking was defined as adding salt at the table. A quadratic weighted kappa statistic was used to evaluate the long-term reliability of adding salt at the table at the baseline and first repeated assessment (mean 4.3 years after baseline) or second repeated assessment (mean 9.0 years after baseline). Moderate reliability was identified with a kappa coefficient of 0.60 for the first repeated assessment and 0.53 for the second (Additional file 1: Table S3).

### Outcome ascertainment

The main outcomes were incident CVD and all-cause mortality, and the secondary outcomes included the main types of CVD (coronary heart disease, heart failure, stroke) and CVD mortality. CVD was identified based on the 9th revision of the International Statistical Classification of Diseases (ICD-9), ICD-10, and Office of Population, Censuses and Surveys-4 (OPCS-4) codes and self-reported data fields with the choice-, disease-, or procedure-specific codes (Additional file 1: Table S1) [16]. We compared the date of the first CVD diagnosis and the baseline assessment visit. Participants with CVD before or at the baseline were ascertained as prevalent CVD cases, while those diagnosed with CVD after baseline were regarded as incident CVD cases. At the time of this analysis, the inpatient record data were available as of March 31, 2021. We censored follow-up at this date or the date of the first incident of CVD or death, whichever came first. As mortality data were available up to 28 February 2021, we censored follow-up at this date or the date of death, whichever occurred first.

### Covariate assessment

Information on sociodemographic factors, lifestyle factors, dietary intake, medical history, and medication use was collected using a touch-screen, self-completed questionnaire at the baseline assessment. Height (m) and body weight (kg) were measured by trained nurses at the baseline. Ethnicity was categorized as White, Mixed, Asian, Black, Chinese, and others. Education was categorized as college or university, vocational, upper secondary, lower secondary, and others. Household income was categorized as <£18,000, £18,000–£30,999, £31,000–£51,999, £52,000–£100,000, and >£100,000. Smoking status was defined as never, former, and current. The frequency and volume of current alcohol consumption were self-reported, and alcohol consumption was considered as a continuous variable. People in the UK are advised not to drink more than 14 units a week (equivalent to 16 g/day) [17]. Socioeconomic status was derived from Townsend deprivation index scores and categorized into quintiles (a higher score denoted a higher degree of deprivation) [18]. Physical activity level over a typical week was self-reported using the validated International Physical Activity Questionnaire, and the total metabolic equivalent of task (MET) in a week was categorized into quintiles [19]. Body mass index (BMI) was calculated as the weight in kilograms (kg) divided by the square of the height in meters (m^2^) and was considered a continuous variable. Medical history (hypertension, diabetes, dyslipidemia, family history of stroke, and heart disease) was classified as yes or no. We constructed a healthy diet score concerning the dietary priorities for cardiometabolic health recommended by the American Heart Association [20]. Definitions of each component of the healthy diet score are shown in Additional file 1: Table S2. The scale of the healthy diet score ranged from 0 to 10, and a higher score denoted a healthier dietary pattern. The healthy diet score was categorized into quintiles [16].

### Statistical analysis

Baseline characteristics were presented as the number (percentage) for categorical variables and the mean (standard deviation) for continuous variables. General linear models were used to evaluate the association between the frequency of adding salt at the table and the estimated 24-h urinary sodium excretion. Multivariable-adjusted Cox proportional hazards models were performed to estimate the hazard ratios (HRs) and 95% confidence intervals (CIs) for the associations of adding salt at the table with incident CVD and mortality. The proportionality assumption was checked by using the Schoenfeld residual test. Three sequential models were used. Model 1 adjusted for age, sex, ethnicity, education, Townsend deprivation index, and household income; model 2 further included smoking status, alcohol consumption, total physical activity, and healthy diet score; model 3 further included BMI, diabetes, dyslipidemia, family history of stroke, and heart disease. Because the formula for calculating estimated 24-h urinary sodium included age, sex, height, and weight [21–23], we did not adjust for age, sex, and BMI in the general linear model. The missing values of covariates were treated as dummy variables.

Subgroup analyses were performed by the following variables: age (<60, ≥60 years), sex (man or woman), education (College or university/Vocational, upper secondary or lower secondary), ethnicity (white, non-white), household income (≤£30,999, >£30,999), Townsend deprivation index (below the median, above the median), smoking status (current, previous, never), alcohol consumption (≤16, >16 g/d), BMI (<30.0, ≥30.0 kg/m^2^), diabetes (yes or no), and dyslipidemia (yes or no). As for hypertension, we stratified it into diagnosed and undiagnosed. Diagnosed hypertension was defined based on ICD-9, ICD-10, a self-reported history of hypertension, or the use of antihypertensive drugs. Given that elevated blood pressure might be the main pathway by which salt affects CVD, rather than a source of reverse causality, screen-detected participants with baseline systolic or diastolic blood pressure ≥140 mmHg or 90 mmHg and those without hypertension were defined as undiagnosed hypertension. The effect modification was assessed by including multiplicative interaction terms with the frequency of adding salt at the table in the models.

Four sensitivity analyses were conducted to test the robustness of the main findings. First, to minimize the potential reverse causation bias, participants who developed CVD or died during the first 2 years of follow-up were excluded and re-ran the main analyses. Second, participants with chronic kidney disease were excluded because they have diminished capacity to excrete sodium and a higher risk of cardiovascular disease [24]. Third, dietary variation may alter the habit of adding salt at the table, and participants whose diets frequently changed week to week were excluded. Fourth, major diet changes before the baseline may contribute to changes in the habit of adding salt at the table, and those who had major dietary changes in the previous 5 years were excluded.

A 2-tailed *P*-value of less than 0.05 was used to determine the statistical significance. Results from subgroup analyses were considered exploratory due to multiple testing. All analyses were performed by SAS version 9.4 software (SAS Institute, USA).

## Results

### Baseline characteristics

Baseline characteristics of the participants according to the frequency of adding salt at the table are displayed in Table 1. Of the 413,109 participants, 187,949 (45.5%) were men, with a mean age (SD) of 55.8 (8.1) years. Overall, 229,593 (55.6%) participants reported never/rarely adding salt at the table, while 183,516 (44.4%) reported adding salt at the table, 116,095 (28.1%) reported sometimes adding salt at the table, 47,703 (11.6%) reported usually adding salt at the table, and 19,718 (4.8%) reported always adding salt at the table. Compared with the reference group (never/rarely adding salt at the table), those who added salt at the table were more likely to be current smokers, drink more alcohol, be more socioeconomically deprived, have a higher level of total physical activity and estimated 24-hour urinary sodium, have a higher prevalence of diabetes and dyslipidemia, be less educated, have lower household income, and have poor diet quality. Additionally, those who added salt at the table had a lower prevalence of hypertension, family history of stroke, and heart disease. After adjustment for covariates, we found a positive association between the frequency of adding salt at the table and the estimated 24-h urinary sodium (Additional file 1: Fig. S2).

**Table 1:** Baseline characteristics of 413,109 participants without cardiovascular disease

Characteristics	Frequency of adding salt at the table			
	Never/rarely	Sometimes	Usually	Always
Mean (SD) age (years)	55.80 (8.08)	55.70 (8.11)	56.26 (8.05)	55.15 (8.26)
Male, *N* (%)	100,439 (43.75)	53,561 (46.14)	24,371 (51.09)	9578 (48.57)
Ethnicity, *N* (%)				
White	218,788 (95.29)	107,962 (92.99)	44,362 (93.00)	17,052 (86.48)
Mixed	1225 (0.53)	799 (0.69)	342 (0.72)	207 (1.05)
Asian	3151 (1.37)	2906 (2.50)	1322 (2.77)	1056 (5.36)
Black	3227 (1.41)	2336 (2.01)	781 (1.64)	738 (3.74)
Chinese	764 (0.33)	372 (0.32)	181 (0.38)	113 (0.57)
Others	1668 (0.73)	1344 (1.16)	546 (1.14)	458 (2.32)
Missing	770 (0.34)	376 (0.32)	169 (0.35)	94 (0.48)
Education, *N* (%)				
College or university	81,589 (35.54)	37,319 (32.15)	14,718 (30.85)	4151 (21.05)
Vocational	25,199 (10.98)	13529 (11.65)	5859 (12.28)	2491 (12.63)
Upper secondary	27,414 (11.94)	12,748 (10.98)	5027 (10.54)	1591 (8.07)
Lower secondary	60,460 (26.33)	31,504 (27.14)	12,901 (27.04)	5496 (27.87)
Others	31,282 (13.62)	18,791 (16.19)	8315 (17.43)	5443 (27.60)
Unknown	3649 (1.59)	2204 (1.90)	883 (1.85)	546 (2.77)
Household income (£)				
<18,000	38,246 (16.66)	20,775 (17.89)	9187 (19.26)	5079 (25.76)
18,000–30,999	48,415 (21.09)	24,866 (21.42)	10,500 (22.01)	4242 (21.51)
31,000–51,999	53,849 (23.45)	26,755 (23.05)	10,606 (22.23)	3682 (18.67)
52,000–100,000	44,504 (19.38)	20,898 (18.00)	8269 (17.33)	2471 (12.53)
>100,000	11,988 (5.22)	5551 (4.78)	2215 (4.64)	569 (2.89)
Missing	32,591 (14.20)	17,250 (14.86)	6926 (14.52)	3675 (18.64)
Smoking status, *N* (%)				
Never	139,329 (60.69)	62,571 (53.90)	22,234 (46.61)	8303 (42.11)
Previous	71,463 (31.13)	40,161 (34.59)	18,071 (37.88)	6754 (34.25)
Current	18,101 (7.88)	12,924 (11.13)	7222 (15.14)	4550 (23.08)
Missing	700 (0.30)	439 (0.38)	176 (0.37)	111 (0.56)
Mean (SD) alcohol consumption (g/day)	15.09 (16.22)	16.65 (17.48)	19.12 (20.00)	20.79 (25.84)
Healthy diet score^a^, mean (SD)	3.21 (1.41)	2.91 (1.37)	2.72 (1.38)	2.55 (1.39)
Mean (SD) total physical activity (MET-min/week)	2650.65 (2656.08)	2662.88 (2733.42)	2670.50 (2793.29)	2921.16 (3223.61)
Median (IQR) Townsend deprivation index	−2.32 (−3.74, 0.13)	−2.07 (−3.60, 0.60)	−1.95 (−3.55, 0.87)	−1.01 (−3.10, 2.32)
Mean (SD) BMI (kg/m^2^)	27.06 (4.68)	27.51 (4.75)	27.72 (4.76)	27.93 (5.05)
Family history of stroke, *N* (%)	62,479 (27.21)	30,719 (26.46)	12,638 (26.49)	4831 (24.50)
Family history of heart disease, *N* (%)	102,214 (44.52)	50,152 (43.20)	20,500 (42.97)	8076 (40.96)
Hypertension, *N* (%)	62,903 (27.40)	28,868 (24.87)	11,558 (24.23)	4769 (24.19)
Diabetes, *N* (%)	11,684 (5.09)	6171 (5.32)	2531 (5.31)	1120 (5.68)
Dyslipidemia, *N* (%)	108,004 (47.04)	56,630 (48.78)	24,212 (50.76)	10,234 (51.90)
Mean (SD) estimated 24-h urinary sodium (mg/day)	4092.82 (1211.57)	4191.76 (1231.19)	4236.76 (1260.74)	4370.51 (1311.85)

Data were expressed as the mean (SD), median (IQR), or *n* (%)

*SD* standard deviation, *MET* metabolic equivalent

^a^Healthy diet score ranged from 0 to 10, and a higher score denoted a healthier dietary pattern

### Associations of adding salt at the table with incident CVD and mortality

During a median follow-up of 12 years (4,757,762 person-years for incident CVD and 4,897,675 person-years for mortality), we documented 37,091 CVD events, including 27,609 coronary heart disease, 8207 heart failure, and 7261 stroke cases, as well as 21,293 deaths, including 4147 CVD deaths. We observed positive associations between the frequency of adding salt at the table and incident CVD and mortality (Table 2). After adjusting for age, sex, and other sociodemographic factors, the HRs (95%CI) of incident CVD across participants who sometimes, usually, and always added salt at the table were 1.07 (1.04–1.09), 1.11 (1.08–1.15), and 1.31 (1.25–1.37); the HRs (95%CI) of all-cause mortality across participants who sometimes, usually, and always added salt at the table were 1.09 (1.05–1.12), 1.17 (1.12–1.22), and 1.45 (1.38–1.54), respectively. In the fully adjusted model, the multivariable-adjusted HRs (95%CI) of incident CVD for participants who sometimes, usually, and always added salt at the table were 1.03 (1.01–1.06), 1.04 (1.01–1.08), and 1.21 (1.16–1.26); the multivariable-adjusted HRs (95%CI) of all-cause mortality for participants who sometimes, usually, and always added salt at the table were 1.03 (1.00–1.07), 1.06 (1.01–1.10), and 1.22 (1.16–1.29), respectively. We further examined the associations between the frequency of adding salt at the table and coronary heart disease, heart failure, stroke, and CVD mortality separately. In the fully adjusted model, compared with participants who never/rarely added salt at the table, participants who always added salt at the table had a higher risk of coronary heart disease (HR: 1.22, 95%CI: 1.16–1.28), heart failure (HR: 1.21, 95%CI: 1.11–1.33), stroke (HR: 1.17, 95%CI: 1.06–1.29), and CVD mortality (HR: 1.19, 95%CI: 1.05–1.35), respectively.

**Table 2:** Associations between adding salt at the table and incident cardiovascular disease and mortality among 413,109 participants

	Frequency of adding salt at the table			
	Never/rarely	Sometimes	Usually	Always
**CVD incidence**				
Cases/person-years	19,140/2,654,769	10,645/1,335,958	4933/544,908	2373/222,127
Model 1	1 (ref)	**1.07 (1.04–1.09)**	**1.11 (1.08–1.15)**	**1.31 (1.25–1.37)**
Model 2	1 (ref)	**1.04 (1.02–1.06)**	**1.06 (1.02–1.09)**	**1.21 (1.16–1.26)**
Model 3	1 (ref)	**1.03 (1.01–1.06)**	**1.04 (1.01–1.08)**	**1.21 (1.16–1.26)**
**Coronary heart disease incidence**				
Cases/person-years	14,212/2,674,176	7910/1,346,554	3700/549,801	1787/224,341
Model 1	1 (ref)	**1.06 (1.03–1.09)**	**1.11 (1.07–1.15)**	**1.31 (1.24–1.37)**
Model 2	1 (ref)	**1.04 (1.01–1.06)**	**1.06 (1.02–1.10)**	**1.22 (1.16–1.28)**
Model 3	1 (ref)	1.03 (1.00–1.06)	**1.05 (1.01–1.09)**	**1.22 (1.16–1.28)**
**Heart failure incidence**				
Cases/person-years	4157/2,730,607	2362/1,378,469	1121/564,201	567/231,282
Model 1	1 (ref)	**1.08 (1.02–1.13)**	**1.13 (1.06–1.20)**	**1.37 (1.25–1.49)**
Model 2	1 (ref)	1.05 (0.99–1.10)	1.06 (0.99–1.13)	**1.23 (1.12–1.35)**
Model 3	1 (ref)	1.02 (0.97–1.08)	1.03 (0.96–1.10)	**1.21 (1.11–1.33)**
**Stroke incidence**				
Cases/person-years	3784/2,729,456	2107/1,377,682	924/564,059	446/231,152
Model 1	1 (ref)	**1.08 (1.02–1.14)**	1.07 (0.99–1.15)	**1.28 (1.16–1.42)**
Model 2	1 (ref)	1.05 (0.99–1.11)	1.01 (0.94–1.09)	**1.16 (1.05**–**1.28)**
Model 3	1 (ref)	1.05 (0.99–1.11)	1.01 (0.94–1.08)	**1.17 (1.06**–**1.29)**
**All-cause mortality**				
Deaths/person-years	10,762/2,725,720	6085/1,376,591	2944/563,957	1502/231,408
Model 1	1 (ref)	**1.09 (1.05**–**1.12)**	**1.17 (1.12**–**1.22)**	**1.45 (1.38**–**1.54)**
Model 2	1 (ref)	**1.04 (1.01**–**1.07)**	**1.06 (1.02**–**1.11)**	**1.23 (1.16**–**1.30)**
Model 3	1 (ref)	**1.03 (1.00**–**1.07)**	**1.06 (1.01**–**1.10)**	**1.22 (1.16**–**1.29)**
**CVD mortality**				
Deaths/person-years	2053/2,725,720	1215/1,376,591	580/563,957	299/231,408
Model 1	1 (ref)	**1.12 (1.04**–**1.20)**	**1.16 (1.05**–**1.27)**	**1.41 (1.25**–**1.60)**
Model 2	1 (ref)	1.07 (1.00–1.15)	1.05 (0.96–1.16)	**1.20 (1.06**–**1.36)**
Model 3	1 (ref)	1.06 (0.98–1.13)	1.04 (0.95–1.14)	**1.19 (1.05**–**1.35)**

Model 1: adjusted for age, sex, ethnicity, education, Townsend deprivation index, household income

Model 2: model 1 + smoking status, alcohol intake, total physical activity, healthy diet score

Model 3: model 2 + body mass index, diabetes, dyslipidemia, family history of stroke, and family history of heart disease

### Secondary analyses

**Fig. 1:**
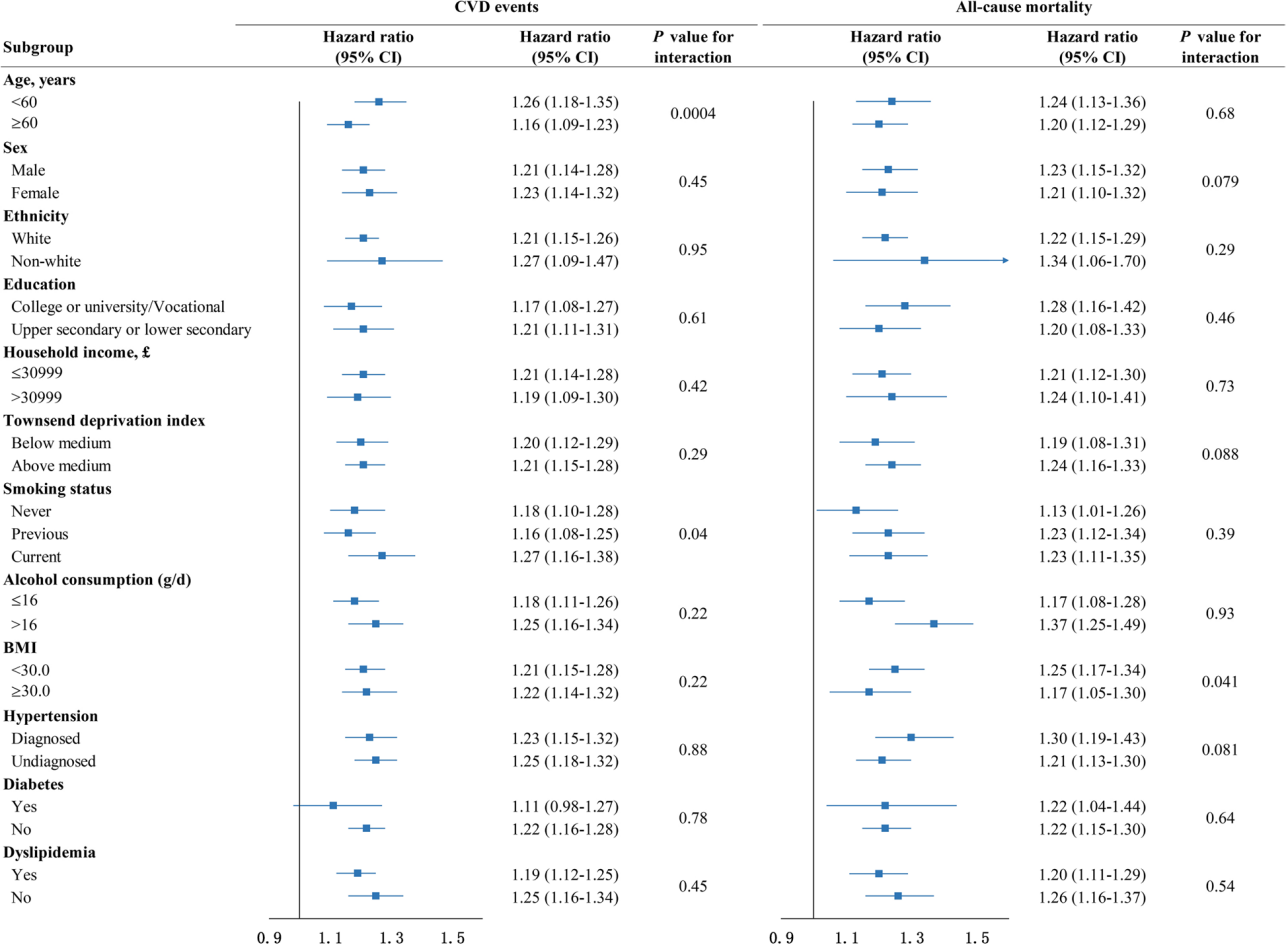
Subgroup analyses of the effects of always adding salt at the table on incident cardiovascular disease and all-cause mortality. Hazard ratios were adjusted for age, sex, ethnicity, education, Townsend deprivation index, household income, smoking status, alcohol intake, total physical activity, healthy diet score, BMI, diabetes, dyslipidemia, family history of stroke, and family history of heart disease. (The stratified variable was not adjusted in the model.) *Healthy diet score ranged from 0 to 10, and a higher score denoted a healthier dietary pattern. Horizontal lines represent 95% confidence intervals

We conducted stratified analyses according to potential risk factors (Fig. 1). Given the existence of multiple comparisons in our analyses, Bonferroni’s correction was intercalated to reduce the likelihood of making a false positive (*P* < 0.004 after Bonferroni's correction). For incident CVD, the association between adding salt at the table and the risk of CVD was stronger among participants aged <60 years (*P* for interaction =0.0004). Sensitivity analyses showed no qualitative change when participants who developed CVD or died during the first 2 years of follow-up were excluded (Additional file 1: Table S4); participants with chronic kidney disease at baseline were excluded (Additional file 1: Table S5); participants whose diet changed frequently every week were removed (Additional file 1: Table S6); or those who had major dietary changes in the last 5 years were excluded (Additional file 1: Table S7).

## Discussion

In this large prospective cohort study, adding salt at the table was significantly associated with a higher risk of incident CVD, CVD mortality, and all-cause mortality. The association was independent of demographic, socioeconomic, lifestyle, and cardiovascular risk factors.

Evidence on the frequency of adding salt at the table and incident CVD and subsequent mortality is limited. A most recent study separated participants who had a habit of adding salt at the table and those who did not use additional salt, founding that adding salt at the table was associated with a 15% higher risk of heart failure [13]. Another study, including 11,742 community older men, reported that always adding salt to food was associated with a 12% higher risk of all-cause mortality [14], which was consistent with our study. Our findings extended these observations to the general population, among whom a positive association between adding salt at the table and incident CVD was first revealed. In addition, ample evidence shows that high salt intake was associated with adverse cardiovascular outcomes and mortality [2, 25–29]. A recent study measured sodium intake in multiple 24-h urine samples, reporting that each daily increase of 1000 mg in sodium excretion (equivalent to 2.5 g salt intake) was associated with an 18% higher CVD risk [2]. In our study, as the frequency with which participants added salt at the table increased, their levels of sodium intake estimated by using urinary sodium excretion increased accordingly. Taken together, our findings emphasized the potential cardiovascular benefits of avoiding or reduction of adding salt at the table and supported the current salt reduction policies.

Several analyses in UK Biobank reported flat associations between the estimated 24-h urinary sodium and CVD [30, 31]. The inconsistency in existing findings may be mainly due to the differences in sodium measurement methods. Both studies used predictive formulas to estimate 24-h urinary sodium as surrogates of sodium intake. However, several confounding variables (e.g., age, sex, weight, height) were used in the formulas, all of which were associated with cardiovascular risk [2]. Interestingly, one study re-adjusted for multiple covariates after removing BMI found that the estimated 24-h urinary sodium was directly and linearly associated with CVD [30]. In this study, we focused on the specific practices for adding salt at the table, rather than the measurement of total salt intake. Adding salt at the table is a common eating habit that reflects a person’s long-standing salt taste preferences and is less likely to be affected by large changes in daily sodium intake [32]. Our study assessed the long-term reliability of adding salt at the table and found a positive association between adding salt at the table and the estimated 24-h urinary sodium. Furthermore, compared to the gold standard for assessing sodium intake (the mean of multiple, nonconsecutive, 24-h urinary sodium), evaluating subjects’ salt habituation in large cohorts may be easier to implement.

Considering the important health implications of sodium intake, WHO and current international dietary guidelines recommend that salt intake for the general population should limit to less than 5 g/day [9] or 6 g/day [10]. However, several studies suggested that when the amount of salt in people’s diet was reduced, they could partially compensate by adding salt to their food at the table [33, 34]. A study found that as the food’s sodium decreased more, the number of participants who added salt after tasting increased. In addition, when sodium was reduced by 30% and accompanied by the reduced-salt label, participants over-compensated for the reduction in salt [34]. Another study also found that more salt was added with increased salt reductions in the meals, and 19% of consumers compensated by adding salt back at the table for full compensation [33]. These studies have shown that when salt is reduced in diets, it may lead to the appearance of adding salt to food at the table, which may ultimately fail to achieve the goal of salt reduction.

Restricting the habit of adding salt to food at the table has been proposed to reduce salt intake [35, 36]. Public health education or removing/replacing salt shakers could also partially reduce salt added at the table [4, 37, 38]. However, the habit of adding salt at the table is prevalent, whether in developed regions [3–5] or in low- and middle-income countries [12, 39–41]. Despite the cardiovascular benefits of reducing salt intake have been increasingly recognized, this has not fully translated into salt reduction action. Moreover, the lack of public awareness of the health benefits derived from not adding salt at the table restricted the implementation of salt reduction actions. Therefore, raising public awareness and implementing salt reduction behavioral intervention is necessary. Our findings first revealed the long-term health hazard of adding salt at the table strongly supported the current public health recommendations and highlighted the benefits of restricting this habit for public health. Achieving and sustaining salt reduction by limiting the habit of adding salt at the table, even by a modest amount, will bring enormous benefits worldwide [35].

This is the first investigation to study the relationship between adding salt at the table and cardiovascular outcomes, with a large sample size and long-term follow-up. Several limitations should be noted. First, adding salt at the table was self-reported, and misclassification of participants was possible. Second, information on salt added at the table was collected at baseline and follow-up visits, but only 10% or fewer participants completed the question during follow-up. Therefore, exploring the associations between changes in adding salt at the table and incident CVD and mortality was not possible. However, this phenomenon would probably bias our data toward the null. Third, our analyses were performed primarily on middle- and old-aged individuals of White British descent, and thus the direct extrapolation of our findings to other populations should be cautious. Fourth, as a prospective cohort study, we cannot exclude the possibility of reverse causation, which may occur when people reduce their sodium intake because of illness or based on medical recommendations. However, our findings were not materially altered when we adjusted for cardiovascular risk factors and excluded participants who developed CVD or died during the first 2 years of follow-up. Additionally, subgroup analyses also indicated that the positive association between adding salt at the table and the risk of CVD was consistent across the subgroup in hypertension. Fifth, although we accounted for a comprehensive list of covariates and conducted several sensitivity analyses, the possibility of residual confounding and potential bias cannot be ruled out. Finally, we cannot assume causality because of the study’s observational nature.

## Conclusions

In this large prospective cohort with nearly half a million UK adults, frequently adding salt at the table was associated with a higher risk of cardiovascular morbidity and mortality. These findings are of great public health implications because of the high prevalence of adding salt at the table in many populations; 44.5% of participants in this study reported habitually adding salt at the table, and 4.8% reported always adding salt at the table. Our study supports the benefits of reducing added salt use at the table in promoting cardiovascular health.

## Supplementary Information

**Additional file 1: Supplementary Materials. Table S1.** Cardiovascular disease definitions in UK Biobank study. **Table S2.** Definition of each component of a healthy diet score. **Table S3.** Cross-tabulation of adding salt at the table at the baseline and the repeated assessment. **Table S4.** Associations between adding salt at the table with incident CVD and mortality after excluding participants who developed CVD or died during the first two years of follow-up. **Table S5.** Associations between adding salt at the table with incident CVD and mortality after excluding participants who were chronic kidney disease at the baseline. **Table S6.** Associations between adding salt at the table with incident CVD and mortality after excluding participants whose diet changed frequently every week. **Table S7.** Associations between adding salt at the table with incident CVD and mortality after excluding participants who had major dietary changes in the previous 5 years. **Figure S1.** Flowchart of eligible population. **Figure S2.** The frequency of adding salt at the table and the estimated 24-h urinary sodium excretion.

## Data Availability

The data of this study can be requested from the UK Biobank (https://www.ukbiobank.ac.uk/).

## Abbreviations

BMI: Body mass index
CI: Confidence interval
CVD: Cardiovascular disease
HR: Hazard ratio
ICD: International Statistical Classification of Diseases
MET: Metabolic equivalent
OPCS-4: Office of Population, Censuses, and Surveys-4
WHO: World Health Organization

## References

[1] Roth GA, Mensah GA, Johnson CO, Addolorato G, Ammirati E, Baddour LM (2020). Global burden of cardiovascular diseases and risk factors, 1990-2019: update from the GBD 2019 study. J Am Coll Cardiol.

[2] Ma Y, He FJ, Sun Q, Yuan C, Kieneker LM, Curhan GC (2022). 24-hour urinary sodium and potassium excretion and cardiovascular risk. N Engl J Med.

[3] Millett C, Laverty AA, Stylianou N, Bibbins-Domingo K, Pape UJ (2012). Impacts of a national strategy to reduce population salt intake in England: serial cross sectional study. PLoS One.

[4] Sutherland J, Edwards P, Shankar B, Dangour AD (2013). Fewer adults add salt at the table after initiation of a national salt campaign in the UK: a repeated cross-sectional analysis. Br J Nutr.

[5] Firestone MJ, Beasley JM, Kwon SC, Ahn J, Trinh-Shevrin C, Yi SS (2017). Asian American dietary sources of sodium and salt behaviors compared with other racial/ethnic groups, NHANES, 2011-2012. Ethn Dis.

[6] Mittelmark MB, Sternberg B (1985). Assessment of salt use at the table: comparison of observed and reported behavior. Am J Public Health.

[7] Cornelio ME, Gallani MC, Godin G, Rodrigues RC, Nadruz W, Mendez RD (2012). Behavioural determinants of salt consumption among hypertensive individuals. J Hum Nutr Diet.

[8] Bhat S, Marklund M, Henry ME, Appel LJ, Croft KD, Neal B (2020). A systematic review of the sources of dietary salt around the world. Adv Nutr (Bethesda, Md).

[9] World Health Organization. Guideline: Sodium intake for adults and children. WHO; 2012. Available from: https://apps.who.int/iris/bitstream/handle/10665/77985/9789241504836_eng.pdf?sequence=1&isAllowed=y. [Cited 2022 Apr 16]. 23658998

[10] Rong S, Liao Y, Zhou J, Yang W, Yang Y (2021). Comparison of dietary guidelines among 96 countries worldwide. Trends Food Sci Technol.

[11] He FJ, Jenner KH, Macgregor GA (2010). WASH-world action on salt and health. Kidney Int.

[12] Webster J, Su'a SA, Ieremia M, Bompoint S, Johnson C, Faeamani G (2016). Salt intakes, knowledge, and behavior in Samoa: monitoring salt-consumption patterns through the World Health Organization’s Surveillance of Noncommunicable Disease Risk Factors (STEPS). J Clin Hypertens (Greenwich).

[13] Dmitrieva NI, Liu D, Wu CO, Boehm M. Middle age serum sodium levels in the upper part of normal range and risk of heart failure. Eur Heart J. 2022. 10.1093/eurheartj/ehac138. 10.1093/eurheartj/ehac138 PMC10263272 35348651

[14] Golledge J, Moxon JV, Jones RE, Hankey GJ, Yeap BB, Flicker L (2015). Reported amount of salt added to food is associated with increased all-cause and cancer-related mortality in older men in a prospective cohort study. J Nutr Health Aging.

[15] Sudlow C, Gallacher J, Allen N, Beral V, Burton P, Danesh J (2015). UK biobank: an open access resource for identifying the causes of a wide range of complex diseases of middle and old age. PLoS Med.

[16] Dai L, Liu M, Chen L (2021). Association of serum 25-hydroxyvitamin D concentrations with all-cause and cause-specific mortality among adult patients with existing cardiovascular disease. Front Nutr.

[17] Service NH. Alcohol units. Available from: https://www.nhs.uk/live-well/alcohol-advice/calculating-alcohol-units/. [Cited 2022 Apr 16].

[18] Foster HME, Celis-Morales CA, Nicholl BI, Petermann-Rocha F, Pell JP, Gill JMR (2018). The effect of socioeconomic deprivation on the association between an extended measurement of unhealthy lifestyle factors and health outcomes: a prospective analysis of the UK Biobank cohort. Lancet Public Health.

[19] Craig CL, Marshall AL, Sjostrom M, Bauman AE, Booth ML, Ainsworth BE (2003). International physical activity questionnaire: 12-country reliability and validity. Med Sci Sports Exerc.

[20] Mozaffarian D (2016). Dietary and policy priorities for cardiovascular disease, diabetes, and obesity: a comprehensive review. Circulation..

[21] Kawasaki T, Itoh K, Uezono K, Sasaki H (1993). A simple method for estimating 24 h urinary sodium and potassium excretion from second morning voiding urine specimen in adults. Clin Exp Pharmacol Physiol.

[22] Mente A, O'Donnell MJ, Dagenais G, Wielgosz A, Lear SA, McQueen MJ (2014). Validation and comparison of three formulae to estimate sodium and potassium excretion from a single morning fasting urine compared to 24-h measures in 11 countries. J Hypertens.

[23] Inker LA, Schmid CH, Tighiouart H, Eckfeldt JH, Feldman HI, Greene T (2012). Estimating glomerular filtration rate from serum creatinine and cystatin C. N Engl J Med.

[24] Mills KT, Chen J, Yang W, Appel LJ, Kusek JW, Alper A (2016). Sodium excretion and the risk of cardiovascular disease in patients with chronic kidney disease. JAMA..

[25] Strazzullo P, D'Elia L, Kandala NB, Cappuccio FP (2009). Salt intake, stroke, and cardiovascular disease: meta-analysis of prospective studies. BMJ..

[26] Poggio R, Gutierrez L, Matta MG, Elorriaga N, Irazola V, Rubinstein A (2015). Daily sodium consumption and CVD mortality in the general population: systematic review and meta-analysis of prospective studies. Public Health Nutr.

[27] Aburto NJ, Ziolkovska A, Hooper L, Elliott P, Cappuccio FP, Meerpohl JJ (2013). Effect of lower sodium intake on health: systematic review and meta-analyses. BMJ..

[28] Cook NR, Appel LJ, Whelton PK (2016). Sodium intake and all-cause mortality over 20 years in the trials of hypertension prevention. J Am Coll Cardiol.

[29] Milajerdi A, Djafarian K, Shab-Bidar S (2019). Dose-response association of dietary sodium intake with all-cause and cardiovascular mortality: a systematic review and meta-analysis of prospective studies. Public Health Nutr.

[30] Elliott P, Muller DC, Schneider-Luftman D, Pazoki R, Evangelou E, Dehghan A (2020). Estimated 24-hour urinary sodium excretion and incident cardiovascular disease and mortality among 398 628 individuals in UK Biobank. Hypertension..

[31] Welsh CE, Welsh P, Jhund P, Delles C, Celis-Morales C, Lewsey JD (2019). Urinary sodium excretion, blood pressure, and risk of future cardiovascular disease and mortality in subjects without prior cardiovascular disease. Hypertension..

[32] Ma H, Xue Q, Wang X, Li X, Franco OH, Li Y (2022). Adding salt to foods and hazard of premature mortality. Eur Heart J.

[33] De Kock HL, Zandstra EH, Sayed N, Wentzel-Viljoen E (2016). Liking, salt taste perception and use of table salt when consuming reduced-salt chicken stews in light of South Africa's new salt regulations. Appetite..

[34] Liem DG, Miremadi F, Zandstra EH, Keast RS (2012). Health labelling can influence taste perception and use of table salt for reduced-sodium products. Public Health Nutr.

[35] He FJ, Tan M, Ma Y, MacGregor GA (2020). Salt reduction to prevent hypertension and cardiovascular disease: JACC state-of-the-art review. J Am Coll Cardiol.

[36] Service NH. Tips for a lower salt diet-Eat well. Available from: https://www.nhs.uk/live-well/eat-well/how-to-eat-a-balanced-diet/tips-for-a-lower-salt-diet/. [Cited 2022 Apr 16].

[37] Goffe L, Wrieden W, Penn L, Hillier-Brown F, Lake AA, Araujo-Soares V (2016). Reducing the salt added to takeaway food: within-subjects comparison of salt delivered by five and 17 holed salt shakers in controlled conditions. PLoS One.

[38] Goffe L, Hillier-Brown F, Doherty A, Wrieden W, Lake AA, Araujo-Soares V (2016). Comparison of sodium content of meals served by independent takeaways using standard versus reduced holed salt shakers: cross-sectional study. Int J Behav Nutr Phys Act.

[39] Bockarie T, Odland ML, Wurie H, Ansumana R, Lamin J, Witham M (2021). Prevalence and socio-demographic associations of diet and physical activity risk-factors for cardiovascular disease in Bo, Sierra Leone. BMC Public Health.

[40] Reyhani P, Azabdaftari F, Ebrahimi-Mamagani M, Asghari-Jafarabadi M, Shokrvash B (2020). The predictors of high dietary salt intake among hypertensive patients in Iran. Int J Hypertens.

[41] Menyanu E, Charlton KE, Ware LJ, Russell J, Biritwum R, Kowal P. Salt use behaviours of Ghanaians and South Africans: a comparative study of knowledge, attitudes and practices. Nutrients. 2017;9(9). 10.3390/nu9090939. 10.3390/nu9090939 PMC5622699 28846641

