# Supplementary material for: Frequency of adding salt at the table and risk of incident cardiovascular disease and all-cause mortality: a prospective cohort study
Source: BMC Med. 2022 Dec 15;20:486. doi: 10.1186/s12916-022-02691-9 (PMC9753015; doi:10.1186/s12916-022-02691-9)
Supplement: Supplementary file 1 — Additional file 1: Supplementary Materials. Table S1. Cardiovascular disease definitions in UK Biobank study. Table S2. Definition of each component of a healthy diet score. Table S3. Cross-tabulation of adding salt at the table at the baseline and the repeated assessment. Table S4. Associations between adding salt at the table with incident CVD and mortality after excluding participants who developed CVD or died during the first two years of follow-up. Table S5. Associations between adding salt at the table with incident CVD and mortality after excluding participants who were chronic kidney disease at the baseline. Table S6. Associations between adding salt at the table with incident CVD and mortality after excluding participants whose diet changed frequently every week. Table S7. Associations between adding salt at the table with incident CVD and mortality after excluding participants who had major dietary changes in the previous 5 years. Figure S1. Flowchart of eligible population. Figure S2. The frequency of adding salt at the table and the estimated 24-h urinary sodium excretion. [file 12916_2022_2691_MOESM1_ESM.doc]

**Supplementary Online Content**

**Frequency of adding salt at the table and risk of incident cardiovascular disease and all-cause mortality: a prospective cohort study**

**Table S1. Cardiovascular disease definitions in UK Biobank study**

**Table S2. Definition of each component of a healthy diet score**

**Table S3. Cross-tabulation of adding salt at the table at the baseline and the repeated assessment**

**Table S4. Associations between adding salt at the table with incident CVD and mortality after excluding participants who developed CVD or died during the first two years of follow-up**

**Table S5. Associations between adding salt at the table with incident CVD and mortality after excluding participants who were chronic kidney disease at the baseline**

**Table S6. Associations between adding salt at the table with incident CVD and mortality after excluding participants whose diet changed frequently every week**

**Table S7. Associations between adding salt at the table with incident CVD and mortality after excluding participants who had major dietary changes in the previous 5 years**

**Fig. S1 Flowchart of eligible population**

**Fig. S2 The frequency of adding salt at the table and the estimated 24-h urinary sodium excretion**

**Table S1. Cardiovascular disease definitions in UK Biobank study**

|  | **ICD-9** | **ICD-10** | **OPCS-4** | **Self-reported field IDs** |
| --- | --- | --- | --- | --- |
| **Coronary Heart Disease** | 410, 411, 412, 413, 414 | I20-25 | K40-K46, K49, K50, K75 | 6150, 3894, 3627, 20002, 20004 |
| **Heart Failure** | 428, 4280, 4281,4289 | I50, I500, I501, I509 |  | 20002 |
| **Stroke** | 430, 431, 434, 4340, 4341, 4349, 436 | I60, I61, I63, I64 | A052-A054, L343, L351, L353 | 4056, 6150, 20002 |

Variable definitions constructed using ICD-9, ICD-10, and OPCS-4 codes as well as self-reported data fields with choice-, disease- or procedure-specific codes between brackets are shown.

Abbreviations: ICD, International Classification of Diseases; OPCS, Office of Population, Censuses and Surveys: Classification of interventions and Procedures

**Table S2. Definition of each component of a healthy diet score**

|  | Goal (1 point) | One serving equals to | Field IDs |
| --- | --- | --- | --- |
| Fruits | ≥ 3 servings/day | 1 piece of fresh fruit  5 pieces of dried fruit | 1309, 1319 |
| Vegetables  (excluding potatoes) | ≥ 3 servings/day | 3 heaped tablespoons | 1289, 1299 |
| Whole grains | ≥ 3 servings/day | 1 slice of whole-grain bread  1 cup of whole-grain cereal | 1438, 1448, 1458, 1468 |
| Vegetable oil | ≥ 2 servings/day | Vegetable oil based spread (Flora Pro-Active/Benecol, Soft (tub) margarine, Olive oil based spread, or Polyunsaturated/sunflower oil based spread) in combination with eating at least 2 slices of bread | 1428, 2654, 1438 |
| Fish | ≥ 2 servings/week | Once/week | 1329, 1339 |
| Dairy | ≥ 2 servings/day | 1 cup/day if consumption any type of milk  1 piece of cheese | 1408, 1418 |
| Refined grains | ≤ 2 servings/day | 1 slice of bread  1 bowl of cereal | 1438, 1448, 1458, 1468 |
| Unprocessed meats | ≤ 2 servings/week | Once/week (including poultry, beef, lamb, and pork) | 1359, 1369, 1379, 1389 |
| Processed meats | ≤ 1 servings/week | Once/week | 1349 |
| Sugar-sweetened beverages | Don’t drink | Only 0 serving was possible here | 6144 |

Field IDs and serving sizes used per diet component in UK Biobank with available data from the general baseline questionnaire. If participants achieved the intake goal, they were considered to have an adequate intake of the diet component.

**Table S3. Cross-tabulation of adding salt at the table at the baseline and the repeated assessment**

|  |  | Baseline | | | | |
| --- | --- | --- | --- | --- | --- | --- |
|  |  | Never/rarely | Sometimes | Usually | Always | Total |
| First repeated assessment (2012-13) | Never/rarely | 8838 | 1367 | 158 | 30 | 10393 |
|  | Sometimes | 1350 | 2504 | 547 | 44 | 4445 |
|  | Usually | 140 | 558 | 871 | 196 | 1765 |
|  | Always | 22 | 73 | 171 | 242 | 508 |
|  | Total | 10350 | 4502 | 1747 | 512 | 17111 |
| Second repeated assessment (2014+) | Never/rarely | 20403 | 3459 | 501 | 102 | 24465 |
|  | Sometimes | 4333 | 6061 | 1490 | 209 | 12093 |
|  | Usually | 610 | 1658 | 1957 | 453 | 4678 |
|  | Always | 84 | 201 | 446 | 528 | 1259 |
|  | Total | 25430 | 11379 | 4394 | 1292 | 42495 |

**Table S4. Associations between adding salt at the table with incident CVD and mortality after excluding participants who developed CVD or died during the first two years of follow-up**

|  | **Frequency of adding salt at the table** | | | |
| --- | --- | --- | --- | --- |
|  | **Never/rarely** | **Sometimes** | **Usually** | **Always** |
| **CVD incidence** | | | | |
| cases/person-years | 17024/2652028 | 9425/1334298 | 4374/544140 | 2087/221748 |
| Model 1 | 1 (ref) | **1.06 (1.04-1.09)** | **1.11 (1.07-1.15)** | **1.30 (1.24-1.36)** |
| Model 2 | 1 (ref) | **1.04 (1.01-1.06)** | **1.06 (1.02-1.10)** | **1.20 (1.15-1.26)** |
| Model 3 | 1 (ref) | **1.03 (1.00-1.05)** | **1.05 (1.01-1.08)** | **1.20 (1.15-1.26)** |
| **Coronary heart disease incidence** | | | | |
| cases/person-years | 12458/2667890 | 6891/1342910 | 3241/548105 | 1550/223534 |
| Model 1 | 1 (ref) | **1.05 (1.02-1.09)** | **1.12 (1.07-1.16)** | **1.30 (1.23-1.37)** |
| Model 2 | 1 (ref) | **1.03 (1.00-1.06)** | **1.07 (1.03-1.11)** | **1.21 (1.15-1.28)** |
| Model 3 | 1 (ref) | 1.02 (0.99-1.05) | **1.06 (1.01-1.10)** | **1.21 (1.15-1.28)** |
| **Heart failure incidence** | | | | |
| cases/person-years | 3794/2709728 | 2147/1366203 | 1036/558741 | 505/228662 |
| Model 1 | 1 (ref) | **1.08 (1.02-1.13)** | **1.15 (1.07-1.23)** | **1.34 (1.22-1.47)** |
| Model 2 | 1 (ref) | 1.04 (0.99-1.10) | **1.08 (1.00-1.15)** | **1.21 (1.10-1.33)** |
| Model 3 | 1 (ref) | 1.02 (0.97-1.08) | 1.05 (0.98-1.12) | **1.19 (1.08-1.31)** |
| **Stroke incidence** | | | | |
| cases/person-years | 3394/2709361 | 1870/1366000 | 814/558931 | 391/228584 |
| Model 1 | 1 (ref) | **1.07 (1.01-1.13)** | 1.05 (0.97-1.14) | **1.27 (1.14-1.41)** |
| Model 2 | 1 (ref) | 1.04 (0.98-1.10) | 1.00 (0.92-1.08) | **1.15 (1.03-1.28)** |
| Model 3 | 1 (ref) | 1.04 (0.98-1.1) | 0.99 (0.92-1.07) | **1.16 (1.04-1.29)** |
| **All-cause mortality** | | | | |
| deaths/person-years | 9976/2702565 | 5562/1363139 | 2687/558015 | 1354/228460 |
| Model 1 | 1 (ref) | **1.08 (1.04-1.11)** | **1.16 (1.11-1.21)** | **1.43 (1.35-1.52)** |
| Model 2 | 1 (ref) | 1.03 (0.99-1.06) | **1.05 (1.01-1.10)** | **1.21 (1.14-1.28)** |
| Model 3 | 1 (ref) | 1.02 (0.99-1.06) | **1.05 (1.00-1.09)** | **1.21 (1.14-1.28)** |
| **CVD mortality** | | | | |
| deaths/person-years | 1834/2702565 | 1067/1363139 | 514/558015 | 258/228460 |
| Model 1 | 1 (ref) | **1.10 (1.02-1.19)** | **1.15 (1.04-1.27)** | **1.38 (1.21-1.58)** |
| Model 2 | 1 (ref) | 1.05 (0.98-1.14) | 1.05 (0.95-1.16) | **1.17 (1.03-1.34)** |
| Model 3 | 1 (ref) | 1.04 (0.96-1.12) | 1.04 (0.94-1.14) | **1.17 (1.02-1.34)** |

Model 1: adjusted for age, sex, ethnicity, education, Townsend deprivation index, household income.
Model 2: model 1 + smoking status, alcohol intake, total physical activities, healthy diet score.
Model 3: model 2 + BMI, diabetes, dyslipidemia, family history of stroke, family history of heart disease.

Abbreviations: BMI: body mass index.

**Table S5. Associations between adding salt at the table with incident CVD and mortality after excluding participants who were chronic kidney disease at the baseline**

|  | **Frequency of adding salt at the table** | | | |
| --- | --- | --- | --- | --- |
|  | **Never/rarely** | **Sometimes** | **Usually** | **Always** |
| **CVD incidence** |  |  |  |  |
| cases/person-years | 18944/2647407 | 10554/1332820 | 4901/543857 | 2357/221661 |
| Model 1 | 1 (ref) | **1.07 (1.04-1.09)** | **1.11 (1.08-1.15)** | **1.31 (1.26-1.37)** |
| Model 2 | 1 (ref) | **1.04 (1.02-1.07)** | **1.06 (1.03-1.09)** | **1.21 (1.16-1.27)** |
| Model 3 | 1 (ref) | **1.03 (1.01-1.06)** | **1.05 (1.01-1.08)** | **1.21 (1.16-1.26)** |
| **Coronary heart disease incidence** | | | | |
| cases/person-years | 14067/2666625 | 7846/1343345 | 3683/548716 | 1775/223870 |
| Model 1 | 1 (ref) | **1.06 (1.03-1.09)** | **1.12 (1.08-1.16)** | **1.31 (1.25-1.38)** |
| Model 2 | 1 (ref) | **1.04 (1.01-1.07)** | **1.07 (1.03-1.11)** | **1.22 (1.16-1.28)** |
| Model 3 | 1 (ref) | **1.03 (1.00-1.06)** | **1.05 (1.02-1.09)** | **1.22 (1.16-1.28)** |
| **Heart failure incidence** | | | | |
| cases/person-years | 4071/2722686 | 2323/1375095 | 1108/563064 | 562/230738 |
| Model 1 | 1 (ref) | **1.08 (1.03-1.14)** | **1.14 (1.06-1.22)** | **1.39 (1.27-1.51)** |
| Model 2 | 1 (ref) | 1.05 (1.00-1.11) | 1.07 (1.00-1.14) | **1.25 (1.14-1.36)** |
| Model 3 | 1 (ref) | 1.03 (0.97-1.08) | 1.04 (0.97-1.11) | **1.22 (1.12-1.34)** |
| **Stroke incidence** | | | | |
| cases/person-years | 3749/2721363 | 2093/1374236 | 914/562931 | 445/230596 |
| Model 1 | 1 (ref) | **1.08 (1.02-1.14)** | 1.07 (0.99-1.15) | **1.29 (1.17-1.43)** |
| Model 2 | 1 (ref) | 1.05 (1.00-1.11) | 1.01 (0.94-1.08) | **1.17 (1.06-1.29)** |
| Model 3 | 1 (ref) | 1.05 (0.99-1.11) | 1.00 (0.93-1.08) | **1.18 (1.06-1.30)** |
| **All-cause mortality** | | | | |
| deaths/person-years | 10627/2717597 | 6007/1373139 | 2919/562805 | 1487/230845 |
| Model 1 | 1 (ref) | **1.09 (1.05-1.12)** | **1.18 (1.13-1.23)** | **1.46 (1.38-1.54)** |
| Model 2 | 1 (ref) | **1.04 (1.01-1.07)** | **1.06 (1.02-1.11)** | **1.23 (1.16-1.30)** |
| Model 3 | 1 (ref) | **1.03 (1.00-1.07)** | **1.06 (1.02-1.10)** | **1.23 (1.16-1.30)** |
| **CVD mortality** | | | | |
| deaths/person-years | 2017/2717597 | 1190/1373139 | 575/562805 | 297/230845 |
| Model 1 | 1 (ref) | 1.11 (1.04-1.20) | **1.16 (1.06-1.28)** | **1.43 (1.26-1.62)** |
| Model 2 | 1 (ref) | 1.07 (0.99-1.15) | 1.06 (0.96-1.17) | **1.21 (1.07-1.37)** |
| Model 3 | 1 (ref) | 1.05 (0.98-1.13) | 1.04 (0.95-1.15) | **1.20 (1.06-1.37)** |

Model 1: adjusted for age, sex, ethnicity, education, Townsend deprivation index, household income.
Model 2: model 1 + smoking status, alcohol intake, total physical activities, healthy diet score.
Model 3: model 2 + BMI, diabetes, dyslipidemia, family history of stroke, family history of heart disease.
Abbreviations: BMI: body mass index.

**Table S6. Associations between adding salt at the table with incident CVD and mortality after excluding participants whose diet changed frequently every week**

|  | **Frequency of adding salt at the table** | | | |
| --- | --- | --- | --- | --- |
|  | **Never/rarely** | **Sometimes** | **Usually** | **Always** |
| **CVD incidence** | | | | |
| cases/person-years | 17652/2469935 | 9629/1225448 | 4443/496167 | 2112/198293 |
| Model 1 | 1 (ref) | **1.06 (1.03-1.09)** | **1.10 (1.07-1.14)** | **1.30 (1.25-1.37)** |
| Model 2 | 1 (ref) | **1.03 (1.01-1.06)** | **1.05 (1.02-1.09)** | **1.20 (1.15-1.26)** |
| Model 3 | 1 (ref) | 1.02 (1.00-1.05) | **1.04 (1.00-1.07)** | **1.20 (1.15-1.26)** |
| **Coronary heart disease incidence** | | | | |
| cases/person-years | 13104/2487956 | 7159/1235087 | 3334/500566 | 1590/200290 |
| Model 1 | 1 (ref) | **1.05 (1.02-1.09)** | **1.11 (1.06-1.15)** | **1.30 (1.23-1.37)** |
| Model 2 | 1 (ref) | **1.03 (1.00-1.06)** | **1.06 (1.02-1.10)** | **1.21 (1.15-1.28)** |
| Model 3 | 1 (ref) | 1.02 (0.99-1.05) | **1.04 (1.01-1.09)** | **1.21 (1.15-1.28)** |
| **Heart failure incidence** | | | | |
| cases/person-years | 3827/2540101 | 2094/1264153 | 1009/513561 | 507/206419 |
| Model 1 | 1 (ref) | 1.05 (1.00-1.11) | **1.12 (1.05-1.20)** | **1.37 (1.25-1.51)** |
| Model 2 | 1 (ref) | 1.02 (0.97-1.08) | 1.05 (0.98-1.13) | **1.23 (1.12-1.35)** |
| Model 3 | 1 (ref) | 1.00 (0.95-1.05) | 1.03 (0.96-1.10) | **1.21 (1.10-1.33)** |
| **Stroke incidence** | | | | |
| cases/person-years | 3500/2538965 | 1925/1263215 | 835/513436 | 393/206251 |
| Model 1 | 1 (ref) | **1.08 (1.02-1.14)** | 1.06 (0.99-1.15) | **1.26 (1.14-1.40)** |
| Model 2 | 1 (ref) | 1.05 (0.99-1.11) | 1.00 (0.93-1.08) | **1.14 (1.03-1.27)** |
| Model 3 | 1 (ref) | 1.05 (0.99-1.11) | 1.00 (0.93-1.08) | **1.15 (1.03-1.28)** |
| **All-cause mortality** | | | | |
| deaths/person-years | 9925/2535471 | 5495/1262228 | 2668/513307 | 1340/206504 |
| Model 1 | 1 (ref) | **1.08 (1.04-1.12)** | **1.17 (1.12-1.22)** | **1.45 (1.37-1.54)** |
| Model 2 | 1 (ref) | 1.03 (1.00-1.07) | **1.06 (1.02-1.11)** | **1.23 (1.16-1.30)** |
| Model 3 | 1 (ref) | 1.03 (0.99-1.06) | **1.06 (1.01-1.10)** | **1.22 (1.15-1.30)** |
| **CVD mortality** | | | | |
| deaths/person-years | 1877/2535471 | 1091/1262228 | 522/513307 | 273/206504 |
| Model 1 | 1 (ref) | **1.11 (1.03-1.20)** | **1.16 (1.05-1.28)** | **1.46 (1.28-1.65)** |
| Model 2 | 1 (ref) | 1.06 (0.99-1.15) | 1.05 (0.95-1.16) | **1.23 (1.08-1.40)** |
| Model 3 | 1 (ref) | 1.05 (0.97-1.13) | 1.04 (0.94-1.15) | **1.23 (1.08-1.40)** |

Model 1: adjusted for age, sex, ethnicity, education, Townsend deprivation index, household income.
Model 2: model 1 + smoking status, alcohol intake, total physical activities, healthy diet score.
Model 3: model 2 + BMI, diabetes, dyslipidemia, family history of stroke, family history of heart disease.

Abbreviations: BMI: body mass index.

**Table S7. Associations between adding salt at the table with incident CVD and mortality after excluding participants who had major dietary changes in the previous 5 years**

|  | **Frequency of adding salt at the table** | | | |
| --- | --- | --- | --- | --- |
|  | **Never/rarely** | **Sometimes** | **Usually** | **Always** |
| **CVD incidence** | | | | |
| cases/person-years | 10869/1649209 | 6207/838579 | 3038/349478 | 1591/145908 |
| Model 1 | 1 (ref) | **1.08 (1.04-1.11)** | **1.15 (1.10-1.19)** | **1.42 (1.35-1.50)** |
| Model 2 | 1 (ref) | **1.04 (1.01-1.08)** | **1.08 (1.03-1.12)** | **1.28 (1.22-1.36)** |
| Model 3 | 1 (ref) | **1.04 (1.00-1.07)** | **1.06 (1.02-1.11)** | **1.27 (1.21-1.34)** |
| **Coronary heart disease incidence** | | | | |
| cases/person-years | 7997/1660468 | 4573/844994 | 2268/352582 | 1180/147557 |
| Model 1 | 1 (ref) | **1.07 (1.03-1.11)** | **1.15 (1.10-1.21)** | **1.41 (1.32-1.50)** |
| Model 2 | 1 (ref) | **1.04 (1.00-1.08)** | **1.08 (1.03-1.13)** | **1.28 (1.20-1.36)** |
| Model 3 | 1 (ref) | 1.03 (0.99-1.07) | **1.07 (1.02-1.12)** | **1.27 (1.19-1.35)** |
| **Heart failure incidence** | | | | |
| cases/person-years | 2285/1692211 | 1306/863571 | 667/361391 | 384/152079 |
| Model 1 | 1 (ref) | 1.07 (1.00-1.14) | **1.16 (1.06-1.27)** | **1.54 (1.38-1.72)** |
| Model 2 | 1 (ref) | 1.02 (0.96-1.10) | 1.06 (0.97-1.16) | **1.34 (1.19-1.50)** |
| Model 3 | 1 (ref) | 1.00 (0.94-1.08) | 1.03 (0.95-1.13) | **1.31 (1.17-1.47)** |
| **Stroke incidence** | | | | |
| cases/person-years | 2219/1691187 | 1296/862531 | 588/361111 | 310/151873 |
| Model 1 | 1 (ref) | **1.12 (1.04-1.19)** | **1.10 (1.01-1.21)** | **1.39 (1.23-1.56)** |
| Model 2 | 1 (ref) | **1.08 (1.01-1.16)** | 1.03 (0.94-1.14) | **1.24 (1.10-1.40)** |
| Model 3 | 1 (ref) | **1.08 (1.01-1.15)** | 1.03 (0.94-1.13) | **1.24 (1.10-1.40)** |
| **All-cause mortality** | | | | |
| deaths/person-years | 6421/1688246 | 3708/861831 | 1841/361091 | 1007/152196 |
| Model 1 | 1 (ref) | **1.09 (1.05-1.14)** | **1.17 (1.11-1.23)** | **1.48 (1.38-1.58)** |
| Model 2 | 1 (ref) | 1.04 (0.99-1.08) | 1.04 (0.99-1.10) | **1.22 (1.14-1.31)** |
| Model 3 | 1 (ref) | 1.03 (0.99-1.07) | 1.04 (0.99-1.10) | **1.22 (1.14-1.30)** |
| **CVD mortality** | | | | |
| deaths/person-years | 1171/1688246 | 730/861831 | 352/361091 | 206/152196 |
| Model 1 | 1 (ref) | **1.15 (1.05-1.27)** | **1.16 (1.03-1.31)** | **1.54 (1.33-1.79)** |
| Model 2 | 1 (ref) | 1.10 (1.00-1.21) | 1.04 (0.92-1.18) | **1.28 (1.10-1.49)** |
| Model 3 | 1 (ref) | 1.08 (0.99-1.19) | 1.03 (0.91-1.16) | **1.27 (1.09-1.48)** |

Model 1: adjusted for age, sex, ethnicity, education, Townsend deprivation index, household income.
Model 2: model 1 + smoking status, alcohol intake, total physical activities, healthy diet score.
Model 3: model 2 + BMI, diabetes, dyslipidemia, family history of stroke, family history of heart disease.
Abbreviations: BMI: body mass index.

**Fig. S1 Flowchart of eligible population**

**
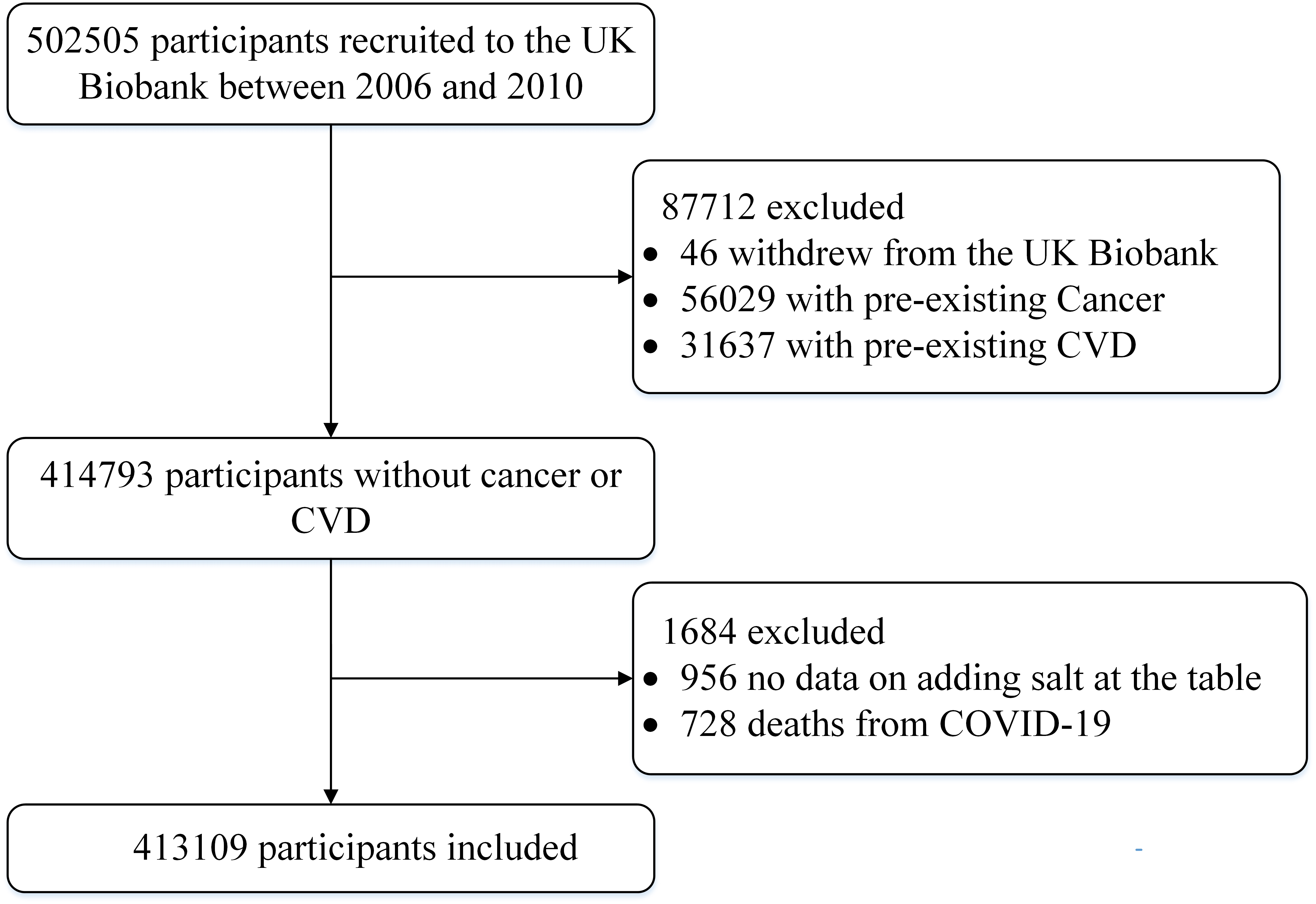
**

**Fig. S2 The frequency of adding salt at the table and the estimated 24-h urinary sodium excretion**

**
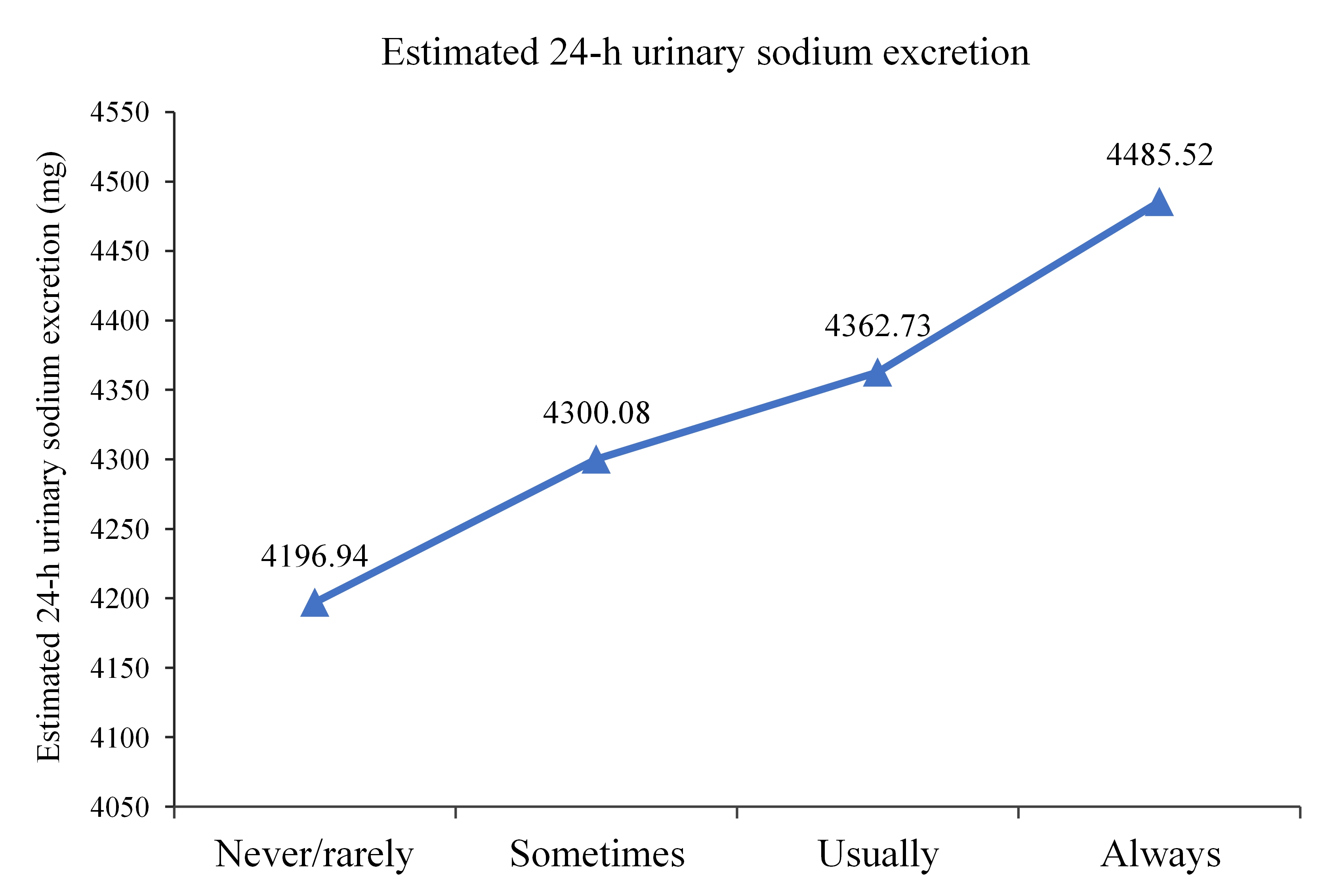
**

Adjusted for ethnicity, education, Townsend deprivation index, household income, smoking status, alcohol intake, total physical activity, healthy diet score, diabetes, dyslipidemia, family history of stroke, and family history of heart disease.
